# Long-Term Endoscopic Gastrostomy Enteral Feeding of Neurosurgical Patients: A Reference Center Experience

**DOI:** 10.3390/biomedicines13071549

**Published:** 2025-06-25

**Authors:** Carolina Palma, Carla Adriana Santos, Ivo Mendes, Francisco Vara-Luiz, Gonçalo Nunes, Irina Mocanu, Cátia Oliveira, Tânia Meira, Marta Brito, Ana Paula Santos, Ana Sofia Gonçalves, Carlos Casimiro, Manuel Cunha e Sá, Jorge Fonseca

**Affiliations:** 1 GENE—Artificial Feeding Team, Gastroenterology Department, Hospital Garcia de Orta, 2805-267 Almada, Portugalfranciscovaraluiz@gmail.com (F.V.-L.); catia.sofia.oliveira@ulsas.min-saude.pt (C.O.); jorgedafonseca@gmail.com (J.F.); 2 Egas Moniz Center for Interdisciplinary Research (CiiEM), Egas Moniz School of Health & Science, 2829-511 Almada, Portugal; 3 Neurosurgery Department, Hospital Garcia de Orta, 2805-267 Almada, Portugal; ana.goncalves@ulsas.min-saude.pt (A.S.G.); carlos.daniel.casimiro@ulsas.min-saude.pt (C.C.);

**Keywords:** percutaneous endoscopic gastrostomy, PEG, neurosurgery, dysphagia, tube feeding, enteral nutrition, stroke, traumatic brain injury, brain tumor

## Abstract

**Background/Objectives:** Nutritional support in neurosurgical patients is challenging due to severe brain injury, neurological disease, or post-surgical complications. This study aimed to assess outcomes of long-term enteral nutrition via endoscopic gastrostomy (PEG) in these patients over a 22-year period. **Methods:** A single-center retrospective (2001–2023) study was conducted on patients referred for PEG. Included patients presented severe traumatic brain injury (TBI), stroke, brain tumor, or other neurosurgical conditions. Demographic, anthropometric, and clinical data were collected. **Results:** A total of 196 patients were included (105 men); 57% were under 65 years. The main diagnoses were stroke (41.8%), TBI (35.2%), and brain tumors (19.9%). The median time from diagnosis to PEG was 94 days. At the time of PEG, only 38.5% were underweight. Outcomes: A total of 132 deaths (75.4%) occurred, while 21 patients resumed oral feeding (10.7%), 22 patients remained PEG-fed (12.6%), and 21 patients were lost to follow-up (10.7%). Most surviving PEG-fed patients had experienced stroke (77%). Median post-PEG survival was 11.5 months and 88% survived >1 month. Higher albumin, transferrin, and cholesterol levels at the time of PEG were associated with longer survival. Albumin (*p* < 0.001) and transferrin (*p* < 0.01) were significantly associated with reduced short-term mortality. **Conclusions:** Despite limited overall survival, reflecting the clinical severity of the diseases, most patients were adequate survivors, and PEG-feeding proved to be appropriate and useful for neurosurgical patients. While most patients had normal-to-high BMI, low serum biomarkers reflected acute illness. Higher serum albumin level was associated with better outcomes, supporting its potential prognostic value.

## 1. Introduction

In neurosurgical departments, patients often face several difficulties with nutritional support due to severe brain injury, neurological disease, or post-surgical complications. While multiple factors may contribute, the most common causes are depressed levels of consciousness or dysphagia resulting from cranial nerve palsies or cortical and brainstem lesions. Assessing the nutritional status of these patients is also challenging, as body mass index (BMI) is difficult to determine in bedridden individuals, and commonly used biomarkers frequently associated with nutrition (albumin, transferrin, and total cholesterol) are nonspecific and influenced by various independent clinical factors, such as acute or chronic inflammation [1,2,3,4,5].

To ensure proper nutrition in these patients, tube feeding is frequently necessary. When long-term enteral nutrition is expected, percutaneous endoscopic gastrostomy (PEG) is the standard approach.

According to the recommendations of the European Society for Clinical Nutrition and Metabolism (ESPEN), PEG is recommended when tube feeding is anticipated for more than four to six weeks [6], and it should not be delayed until severe malnutrition has developed. Worldwide, PEG is one of the most performed endoscopic procedures, and its safety and potential complications are well-documented [7,8,9,10,11]. Before placing a gastrostomy feeding tube, each case should be carefully assessed on an individual basis, considering the patient’s clinical condition, diagnosis, prognosis, anticipated impact on quality of life, and personal preferences [6,7,12].

The number of PEG procedures has been steadily increasing worldwide, particularly among elderly patients, reflecting demographic shifts and advances in endoscopic techniques and device design [13,14]. In the general population, the most common indications for PEG include neurodegenerative conditions such as stroke or amyotrophic lateral sclerosis, as well as head and neck or esophageal cancers. In most of these cases, malnutrition is already present at the time of gastrostomy [6,15].

Our multidisciplinary Artificial Nutrition Team (GENE) has over 26 years of experience, having managed more than 1500 gastrostomy procedures with ongoing outpatient follow-up care at our center [15]. The team, composed of gastroenterologists, general surgeons, nurses, dietitians, and pharmacists, works collaboratively to ensure comprehensive and coordinated care. Additionally, our hospital houses the largest neurosurgery department in southern Portugal and serves as a national reference center for brain tumor treatment. The nutritional support of neurosurgical patients is frequently managed by the Artificial Nutrition Team, benefiting from our extensive expertise in enteral nutrition. Over time, we have observed that neurosurgical patients undergoing PEG may represent a distinct subpopulation with diverse characteristics.

Despite the clinical relevance of PEG in this setting, the literature specifically addressing its use in neurosurgical patients remains limited. To the best of our knowledge, only a few studies have addressed this topic [10,16,17]. In order to increase awareness of the outcomes and usefulness of PEG-feeding in this population, we present and analyze a cohort of patients with neurosurgical conditions who underwent PEG at our center. By examining patient demographics, nutritional status, indications for PEG, and clinical outcomes, we aim to provide valuable insights into the role of PEG in supporting the nutritional needs of this population and to identify factors associated with patient outcome.

## 2. Materials and Methods

### 2.1. Study Design

The investigators conducted a single center, retrospective analysis of neurosurgical patients who were referred for PEG to the Artificial Nutrition Team of Hospital Garcia de Orta, a tertiary hospital in the Lisbon metropolitan area, between 1 January 2001 and 31 December 2023.

### 2.2. Patients

The inclusion criteria encompassed adult patients who suffered severe traumatic brain injury (TBI), hemorrhagic stroke or ischemic stroke with hemorrhagic transformation that required neurosurgical approach, brain tumor patients who underwent neurosurgery, patients with other tumors that required neurosurgical intervention, other conditions such as hydrocephalus, and patients who required PEG for nutritional support. Patients who suffered hemorrhagic stroke without neurosurgical intervention and ischemic stroke patients were excluded from the study. The following demographic data were collected: gender, age, and age group (younger than 65 years, or 65 years or older). 

PEG procedure was decided on a case-by-case basis by the Artificial Nutrition Team in collaboration with the neurosurgical team. It was considered when dysphagia was expected to last over 4–6 weeks and expected survival was adequate. Patients were assessed for clinical condition, neurological prognosis, functional status, and nutritional state. PEG was avoided in cases of poor short-term prognosis, active sepsis, or uncorrectable coagulopathy. Informed consent was obtained from the patient or legal surrogate.

This retrospective cohort included all eligible cases over a 23-year period (2001–2023). All patients meeting criteria were included, ensuring a convenience sampling with maximum representativeness of neurosurgical patients in the 21st century.

### 2.3. Clinical Outcome

To evaluate clinical outcomes, we collected the date of diagnosis, date of the endoscopic gastrostomy procedure and the date of death. The patient status at the end of the study period was classified into four categories: alive and still PEG-fed, alive after PEG tube removal (resumed oral feeding), deceased or lost to follow-up. We then calculated the survival time for all patients, using the date of PEG and the date of death, or 31 December 2023 if the patient was still alive at the end of the study period. Patients were classified as short survivors if the survival period was less than four weeks—the minimum adequate period for gastrostomy, according to guidelines [6,18,19]—or adequate survivors if they survived for four weeks or longer. The time span from diagnosis until PEG procedure was also assessed.

### 2.4. Nutritional Status

To assess nutritional status, BMI was obtained at the moment of the endoscopic gastrostomy using the equation height/weight^2^. If these measurements were not easily obtained due to patients having mobility limitations, the BMI was estimated using the regression equations of Powell–Tuck and Hennessy based on measurement of mid-upper-arm circumference (MUAC) [20]. MUAC was measured in centimeters using a flexible measuring tape wrapped around the upper-arm, halfway between the olecranon and the acromion process. These equations are proven to provide a reliable BMI estimation in gastrostomy patients [21,22]. Patients younger than 65 years of age were categorized according to BMI as underweight (BMI ≤ 18.5 kg/m^2^), normal (BMI 18.5–24.9 kg/m^2^), overweight (BMI 25–29.9 kg/m^2^), or obese (BMI ≥ 30 kg/m^2^). Those aged 65 years or older were categorized as underweight (BMI ≤ 22 kg/m^2^), normal (BMI 22–26.9 kg/m^2^), overweight (BMI 27–29.9 kg/m^2^), or obese (BMI ≥ 30 kg/m^2^).

### 2.5. Laboratory Evaluation

A blood sample was collected at the moment of the gastrostomy and sent for laboratory evaluation. Data were recorded regarding serum albumin, serum transferrin, and serum total cholesterol. Values were then classified as low if albumin <3.5 g/dL, transferrin <200 mg/dL, and total cholesterol <160 mg/dL, these being suggestive of poor prognosis and/or malnutrition.

### 2.6. Statistical Analysis

Statistical analysis was performed using the Statistical Package for Social Sciences (IBM SPSS^®^ Statistics, version 28.0). Continuous variables were expressed as means and standard deviations if normally distributed, or medians and interquartile ranges if not. Categorical variables were expressed as total and relative frequencies. Normality was assessed using the Shapiro–Wilk test. Survival was plotted using the Kaplan–Meier survival analysis and differences between groups were determined with the Log-rank test. A Cox regression model was used for multivariate analysis of survival time. The T-test and the Kruskal–Wallis test were used to compare two independent samples, depending on whether the continuous variables followed a normal distribution or did not, respectively. The relationship between categorical variables was investigated using the Chi-square test. The Pearson correlation test was used to assess the strength and direction of the relationship between two continuous variables. A *p*-value < 0.05 was considered statistically significant.

## 3. Results

### 3.1. Patients

**Figure 1:**
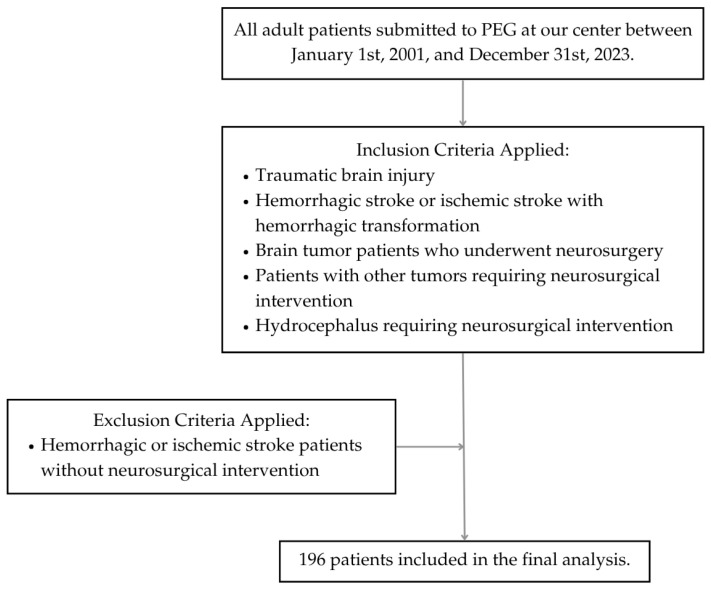
Flow diagram of patient selection.

A total of 196 patients met the inclusion criteria (Figure 1). This cohort comprised 105 men and 91 women, ranging in age from 18 to 87 years (mean age 58.4). Among them, 84 (42.9%) were aged 65 years or older, while 112 (57.1%) were younger than 65 years. The most common condition was stroke requiring neurosurgical intervention, hemorrhagic or ischemic with hemorrhagic transformation (*n* = 82, 41.8%), followed by TBI (*n* = 69, 35.2%) and brain tumors (*n* = 39, 19.9%). Other underlying disorders included hydrocephalus (*n* = 3, 1.5%), 2 cases of cervical chordomas (1%), and 1 case of recurrent meningitis due to a malformation at the base of the skull (0.5%) (Table 1).

The median period from diagnosis of underlying condition until PEG was 94 days. Brain tumor patients and cervical chordoma patients presented a longer median time until the gastrostomy (109.7 and 169.5, respectively), when compared with stroke or TBI patients (93.75 and 77.06, respectively) (*p* < 0.01).

### 3.2. Nutritional Status

**Figure 2:**
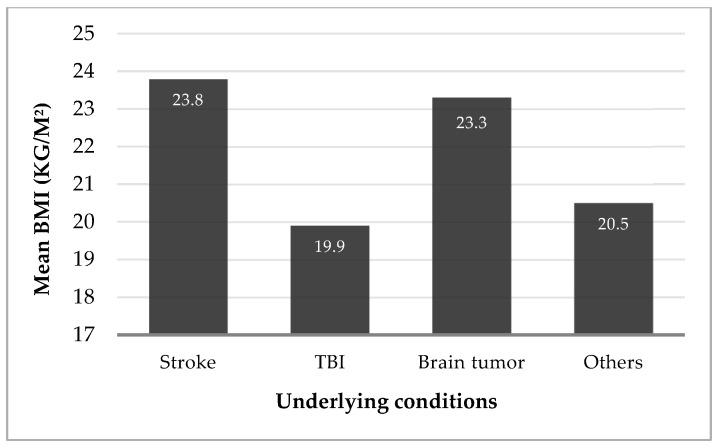
Mean body mass index (BMI) in kg/m^2^ by underlying conditions.

At the date of the gastrostomy, BMI of 169 patients was assessed, using the equation weight/height^2^ in 110 patients, and estimated by the Powell–Tuck and Hennessy regression equations in 59 patients. Of these 169 patients, 65 were underweight (38.5%), 70 were classified as having normal weight (41.4%), 19 were overweight (11.2%), and 15 were obese (8.9%). When analyzing underweight patients based on their underlying conditions, the majority were found to be either TBI patients (46.2%) or stroke patients (30.8%), as shown in Figure 2. A one-way ANOVA was conducted to compare BMI across four diagnostic groups: stroke, TBI, brain tumor, and other neurosurgical conditions, showing a significant difference in BMI across underlying conditions (*F*(3, 104) = 5.73, *p* = 0.01). Post hoc analysis using Tukey’s HSD revealed a statistically significant difference in BMI between stroke and TBI patients (mean difference = 3.90 kg/m^2^, *p* = 0.001, 95% CI [1.25, 6.56]) and between TBI and brain tumor patients (mean difference = −3.43 kg/m^2^, *p* = 0.022, 95% CI [0.37, 6.50]). No other group comparisons reached statistical significance.

We also evaluated baseline levels of serum albumin, transferrin, and total cholesterol. Data were available for 174 patients for albumin, 168 for transferrin, and 166 for total cholesterol. Of these, 95 patients (54.6%) presented low albumin, 102 (61.1%) presented low transferrin, and 75 (45.2%) presented low total cholesterol at the time of PEG.

### 3.3. Clinical Outcomes

At the end of the study period, 21 patients were lost to follow-up. From the remaining 175 patients, 132 died (75.4%), 22 were alive and still PEG-fed (12.6%), and 21 resumed oral feeding and the tube was removed (12%). 

No major PEG-related complications were reported. During follow-up, 13 patients developed superficial stoma infections, which were managed medically with antibiotics administered through the PEG tube. One case of buried bumper syndrome required endoscopic removal and replacement of the gastrostomy tube.

**Figure 3:**
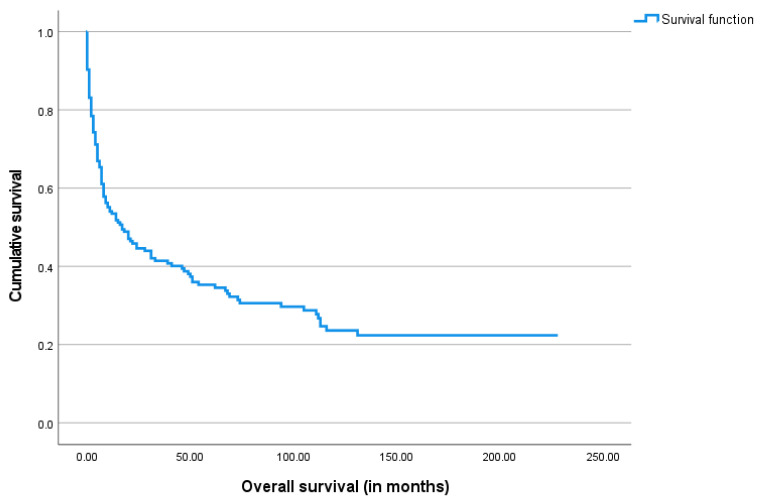
The Kaplan–Meier curve of overall survival for the study cohort.

**Figure 4:**
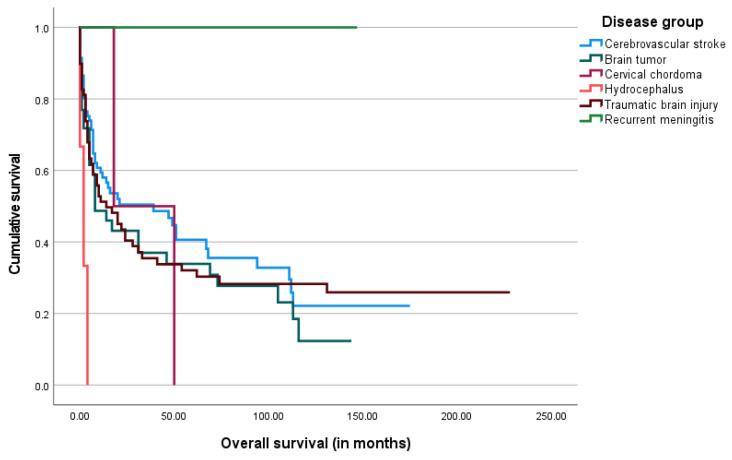
The Kaplan–Meier curve of overall survival by underlying disease group.

**Figure 5:**
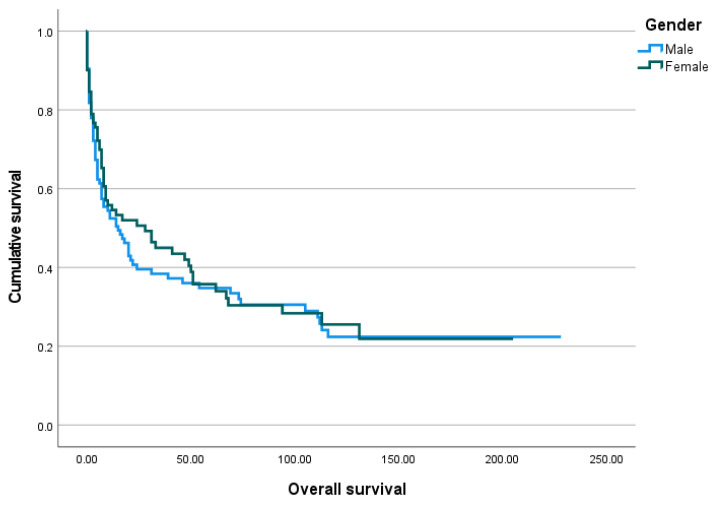
The Kaplan–Meier curve of overall survival by gender.

**Figure 6:**
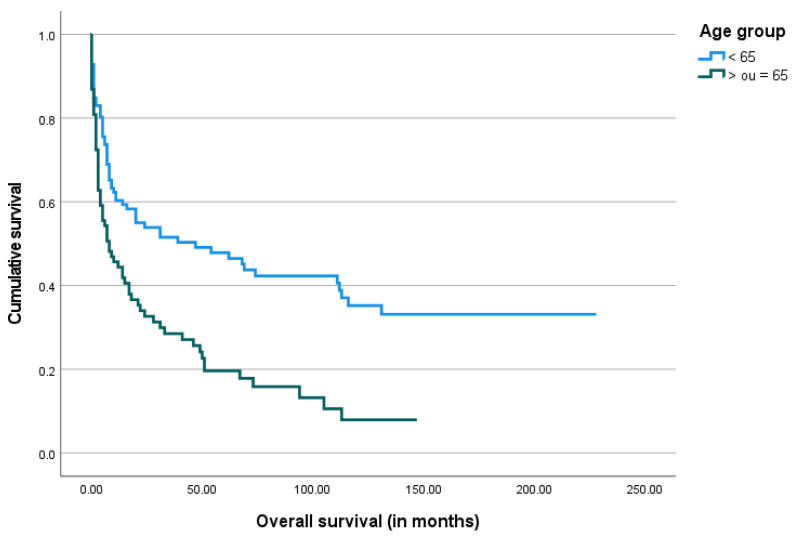
The Kaplan–Meier curve of overall survival by age group.

The median overall survival after the gastrostomy procedure was 11.5 months, ranging from 0 days to 228 months. The Kaplan–Meier survival analysis was performed and is shown below in Figure 3, Figure 4, Figure 5 and Figure 6.

Of the 21 patients who, by the end of the study, had the PEG tube removed and were able to resume oral feeding, 9 were stroke patients (43%), 6 were TBI patients (28.6%), and 5 were brain tumor patients (24%). Additionally, there was one case involving a patient with recurrent meningitis.

Among the 22 patients who were still alive and PEG-fed by the end of the study period, the majority (*n* = 17) were stroke patients, accounting for 77%. The remaining were 3 brain tumor patients (14%) and 2 TBI patients (9%). There were no statistically significant differences between the outcome and the underlying disorder.

Of the total 175 patients, 154 (88%) were classified as adequate survivors, while 21 (12%) were classified as short survivors. When analyzing short survivors, the most common underlying disorders were hemorrhagic stroke (*n* = 8, 38%) and TBI (*n* = 7, 33%), followed by brain tumors (*n* = 5, 24%). These patients were older (mean age 67.1 years) than adequate survivors (mean age 60 years), a statistically significant difference (*p* = 0.03). There were no differences regarding gender. At the day of the gastrostomy procedure, short survivors presented lower levels of albumin, transferrin, and total cholesterol, as well as BMI. However, these differences were only statistically significant for albumin (*p* < 0.01) and transferrin levels (*p* < 0.01).

**Figure 7:**
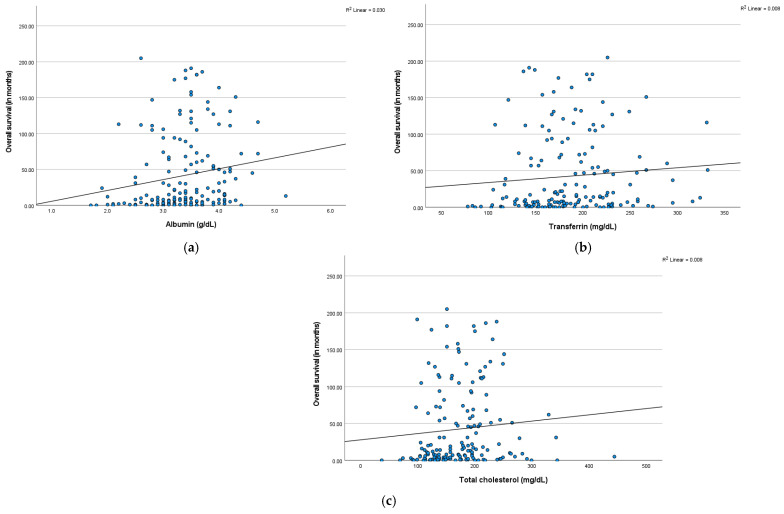
Scatter plots of overall survival versus (**a**) serum albumin levels (in g/dL), (**b**) serum transferrin levels (in mg/dL), and (**c**) serum total cholesterol levels (in mg/dL), with linear regression lines.

Across the entire cohort, initial levels of albumin (r = 0.2), transferrin (r = 0.1), and total cholesterol (r = 0.1) presented a weak positive correlation with patient survival, as shown by the Pearson correlation test. The respective scatter plots are shown below in Figure 7a–c.

A multivariate analysis was conducted using a Cox regression model to evaluate the impact of underlying disease, age, BMI and levels of albumin, transferrin, and total cholesterol on survival time, as shown in Table 2. Covariates were selected based on clinical relevance and prior evidence. Age, BMI, and serum levels of albumin, transferrin, and total cholesterol were modeled as continuous variables. The model’s overall significance was confirmed by the Omnibus Test of Model Coefficients, which showed a statistically significant result (χ^2^ (8) = 37.84, *p* < 0.001).

Regarding the underlying disease category, only stroke, TBI, and brain tumors were included, as other conditions were not sufficiently represented to ensure model stability. Compared to stroke, brain tumors were associated with a 25% higher risk of death, while TBI showed a 48% increased risk; however, neither result was statistically significant (*p* > 0.05). A higher BMI was linked to a 6% reduction in mortality risk, though this did not reach statistical significance (*p* = 0.08). In contrast, older age was a significant predictor of increased mortality risk, with a 5% rise in hazard per additional year of age (*p* < 0.01). Among biochemical markers, higher albumin levels were significantly associated with improved survival, reducing hazard risk by 60% (*p* = 0.03). This means that for each unit increase in albumin (g/dL), the risk of death decreased by approximately 60%. Transferrin and total cholesterol levels were not significantly correlated with survival.

## 4. Discussion

Neurosurgical patients present a high risk for malnutrition and aspiration due to persistently depressed levels of consciousness and frequent dysphagia. When long-term enteral nutrition is required, PEG is the most adequate approach, and it is preferred over nasogastric tube due to less patient discomfort, fewer risk of tube dislodgement or obstruction, and other complications like nasogastric bleeding [9,23].

Our study included 196 neurosurgical PEG-fed patients. In our cohort, cerebrovascular disease, including hemorrhagic or ischemic stroke with hemorrhagic transformation, accounted for 41.6% of cases, TBI for 35.2%, and brain tumors for 19.9%. The most common underlying conditions requiring PEG in our experience were consistent with those reported in other studies of neurosurgical patients [9,16]: the average age at the time of the PEG procedure was lower than reported in other large global PEG cohorts [15,24], which may be attributed to the underlying conditions in our patients, particularly the high prevalence of TBI, which is frequent in younger adults.

PEG was performed at a median of 94 days after diagnosis. In acute neurosurgical patients, such as the majority of our cohort, the hope for neurological recovery may contribute to postponing the decision to initiate long-term enteral feeding via PEG. Patients with brain tumors experienced longer intervals compared to those with other conditions. Review of clinical records revealed that, in several cases, PEG was performed only after tumor recurrence or following surgical procedures that resulted in neurological deterioration, which likely explains the longer intervals from diagnosis to PEG tube placement observed in this group.

Although over half of the patients presented with a normal or elevated BMI at the time of PEG placement, many exhibited low serum albumin, transferrin, and total cholesterol levels. This discrepancy likely reflects the acute nature of their conditions, as albumin and transferrin are negative acute-phase reactants that decrease during inflammation or metabolic stress. Most patients did not appear overtly malnourished based on BMI alone, which contrasts with findings from studies involving other underlying conditions, where lower BMI values at the time of PEG are more frequently reported [15,25,26]. Nonetheless, BMI alone is an unreliable nutritional marker in acutely ill or bedridden patients, as it can be affected by fluid retention, sarcopenia, and systemic inflammation [27,28]. Among patients with low BMI, the majority were TBI or stroke patients. Given the higher representation of these two categories, we further analyzed BMI differences across all groups and found that TBI patients were more frequently malnourished at the time of PEG tube insertion compared to brain tumor or stroke patients. This may be due to the more severe nature of TBI and its associated hypermetabolic state, which increases nutritional demands.

Regarding clinical outcomes, most patients in our sample (75.4%) died by the end of the study period, with a median overall survival of 11.5 months. This high mortality rate reflects the severity of their underlying conditions. Only a small proportion (12%) was alive and had successfully resumed oral feeding, allowing for PEG tube removal. Among the surviving patients who remained PEG-fed, the majority (77%) were stroke patients, who often sustain lesions that are irreversible yet non-progressive over many years.

Most patients (88%) survived more than four weeks after gastrostomy and were therefore classified as adequate survivors, supporting the overall adequacy of the procedure. Only 20 patients (11.4%) died within the first month of PEG-feeding. No significant differences were found in the distribution of underlying diseases between short and adequate survivors.

Given the high mortality rates and severity of underlying conditions in this population, the decision to proceed with PEG often involves complex clinical and ethical considerations. Careful assessment of prognosis, expected quality of life, and patient or surrogate preferences is essential to ensure that interventions align with individualized goals of care. Multidisciplinary is crucial for navigating these challenges and supporting shared decision-making.

According to our results, survival time tended to be longer in patients with higher BMI, serum albumin, transferrin, and total cholesterol levels. Among these variables, only serum albumin presented a significant impact on overall survival. Nevertheless, both high albumin and transferrin levels showed a statistically significant positive effect on decreasing the risk of short survival. In contrast, older age was found to have a negative impact on survival. Regarding the impact of underlying diagnosis, although patients with brain tumors and TBI showed a trend toward higher mortality compared to those with stroke, these differences were not statistically significant. This may reflect a true lack of association or limited power to detect modest effect sizes, particularly in the smaller brain tumor subgroup.

Our study presented some limitations. As a retrospective study, it is subject to sample selection bias. Several clinical reports were incomplete, and there was some lack of patient follow-up, particularly during the COVID-19 pandemic, which prevented a complete analysis of the impact of PEG. Although the study spans over two decades, the criteria for PEG placement and definitions of prolonged dysphagia have remained consistent. While procedural techniques and devices have improved, the core indications, methods, and data collection practices have not significantly changed, minimizing the risk of temporal bias. Despite these limitations, we present an important evaluation of endoscopic gastrostomy in neurosurgical patients. Our findings highlight the role of PEG as a viable method for long-term enteral nutrition in this high-risk population, with adequate survival rates that support its clinical use. Also, the present study identifies some outcome associated markers that may help clinical decisions. Future prospective studies with standardized data collection and longer follow-up periods may better assess long-term outcomes, complications, and quality of life associated with PEG in neurosurgical patients. Multicenter comparisons could improve generalizability. Given the unique challenges faced by neurosurgical patients—such as cognitive impairment and prolonged disability—incorporating patient-reported outcomes and caregiver perspectives will be especially important to fully understand the impact of PEG on quality of life and functional recovery.

## 5. Conclusions

PEG is a useful method for long-term enteral nutrition in neurosurgical patients, when oral intake is compromised. Stroke, TBI, and brain tumors remain the primary indications, sometimes occurring at a younger age compared to the general population of patients requiring PEG. Although overall survival is limited, reflecting the severity of the underlying conditions, most patients survived beyond one month, supporting the adequacy of patient selection for gastrostomy feeding. Higher serum albumin levels were associated with longer survival, suggesting its potential role as a prognostic biomarker in this population. Careful patient selection, timely intervention, and consideration of nutritional status may help optimize outcomes for neurosurgical patients requiring PEG. Future studies should focus on standardized data, patient-reported outcomes, and the unique needs of neurosurgical patients to further optimize care and quality of life.

## Figures and Tables

**Table 1:** Distribution of underlying conditions and the respective percentage.

Underlying Conditions	Total: *n*	Percentage
All conditions	196	100.0%
Stroke	82	41.8%
Traumatic brain injury	69	35.2%
Brain tumor	39	19.9%
Hydrocephalus	3	1.5%
Cervical chordoma	2	1.0%
Recurrent meningitis	1	0.5%

**Table 2:** Results from the multivariate Cox regression analysis evaluating the impact of underlying disease, age, BMI (kg/m^2^), and serum levels of albumin (g/dL), transferrin (mg/dL), and total cholesterol (mg/dL) on survival time.

			95.0% Confidence Interval to HR	
	Sig.	Hazard Ratio (HR)	Lower Bound	Upper Bound
Stroke vs. Brain tumor	0.555	1.252	0.594	2.640
Stroke vs. TBI	0.372	1.482	0.625	3.516
BMI	0.080	0.939	0.876	1.007
Serum albumin	0.025	0.398	0.178	0.890
Serum transferrin	0.703	1.002	0.993	1.011
Serum total cholesterol	0.725	0.999	0.993	1.005
Gender (male vs. female)	0.832	0.922	0.436	1.951
Age	<0.001	1.047	1.025	1.069

## Data Availability

The data presented in this study are available upon request, due to ethical reasons.

## Abbreviations

The following abbreviations are used in this manuscript:

BMI	Body mass index
MUAC	Mid-upper-arm circumference
TBI	Traumatic brain injury
PEG	Percutaneous endoscopic gastrostomy
